# Modeling the geographical distributions of *Chordodes formosanus* and its mantis hosts in Taiwan, with considerations for their niche overlaps

**DOI:** 10.1002/ece3.9546

**Published:** 2022-11-27

**Authors:** Mattia De Vivo, Jen‐Pan Huang

**Affiliations:** ^1^ Biodiversity Research Center Academia Sinica Taipei Taiwan; ^2^ Department of Life Science National Taiwan Normal University Taipei Taiwan; ^3^ Biodiversity Program, Taiwan International Graduate Program Academia Sinica and National Taiwan Normal University Taipei Taiwan

**Keywords:** Maxent, Nematomorpha, parasitism, species distribution models, Taiwan

## Abstract

Species distribution models (SDMs) have conventionally been used for evaluating the distribution of individual species, but they can also be used, through comparing different SDMs, to evaluate the geographic similarity between taxa. In this study, we used a parasite and host system to infer the geographic overlaps between species with tight biological interaction, for example, parasites and their obligate host. Specifically, we used the horsehair worm *Chordodes formosanus* and its three mantis hosts to study the extent of niche overlap. We retrieved presence points for the host species and the parasite, and then we built SDMs with MaxEnt implemented in ENMeval using selected bioclim variables (based on variance inflation factor values) at 30s scale. The models showed that the hosts and parasite do not occur in the high elevation areas in Taiwan, which is expected based on their biology. Interestingly, the predicted parasite distribution included areas without collection records, implying local extinction or sampling bias. We subsequently evaluated niche overlap between hosts and the parasite according to five similarity indices (Schoener's *D*, *I* statistic, relative rank, Pearson correlation coefficient, and the rank correlation coefficient rho). Our models showed a high similarity of SDM predictions between hosts and the parasite. There were differences among metrics for which host shared the highest similarity with the parasite, but the majority of the results indicated that the Japanese boxing mantis had the highest niche similarity with the horsehair worm. The choice of the niche overlap metric to use can uncover information on the parasite's ecology, which can be important for endangered species. SDMs are reliable tools for host and parasite conservation management and could help improve our understanding of parasite biology and ecology.

## Graphical abstract

Use of SDMs to check on the distribution of a parasite and its hosts. SDMs are reliable tools for host and parasite conservation management.

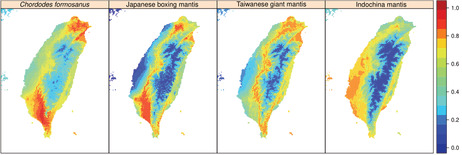

## 1 INTRODUCTION

Species distribution models (SDMs) are an important tool for not only understanding the distribution of targeted species, but also for inferring the changes in biodiversity patterns across space and time (i.e., Lourenço‐de‐Moraes et al., 2019; Zwiener et al., 2017). However, it is still difficult to determine whether biotic interactions might be responsible for shaping the distribution of species (Anderson, 2017; Dormann et al., 2018) although several studies have attempted to do so (i.e., Chang et al., 2021; de Araújo et al., 2014; Kass et al., 2020; Palacio & Girini, 2018; Preuss & Padial, 2021; Simões & Peterson, 2018). Another way to evaluate such interactions is to use niche overlap, defined as the similarity between or among several habitat suitability models (Warren et al., 2008). This has been useful for determining overlaps between species that are supposed to have tight biological interactions, such as prey–predator (e.g., Holt et al., 2018) and host–parasite (e.g., Filz & Schmitt, 2015; Maher et al., 2010; Waltari & Perkins, 2010). Niche overlap can be especially useful for evaluating the latter system, given that the distribution of parasites can be shaped by their obligate hosts (especially for parasites with low dispersal ability) due to the biological and ecological dependency on their hosts (Auld & Tinsley, 2015; Waltari & Perkins, 2010).

Parasites are a major part the ecosystems, as evident from their species diversity (Poulin & Morand, 2004), biomass (Kuris et al., 2008), and importance in food webs (Amundsen et al., 2009; Dougherty et al., 2016; Lafferty et al., 2008; Sato et al., 2008, 2011, 2012). Identifying factors that impact their distribution can be helpful in several ways. For example, these factors help us understand the range and specificity of the parasites by comparing their distribution to their hosts (Filz & Schmitt, 2015; Maher et al., 2010; Waltari & Perkins, 2010), modeling the distribution of a single parasite for inferring the distribution pattern of related species (Haverkost et al., 2010) or using the predicted distribution for sampling surveys (Glass et al., 2021; Harkins et al., 2016) and understand where sampling effort should increase (Pappalardo et al., 2020). In addition, parasite conservation is becoming increasingly important (Carlson et al., 2017; Dobson et al., 2008; Dougherty et al., 2016; Dunn et al., 2009; Gómez & Nichols, 2013; Koh et al., 2004; Kwak et al., 2020) and at least one worldwide conservation plan has been proposed (Carlson et al., 2020). The worldwide conservation plan of parasites is leading to a paradigm shift in conservation biology, because parasites are often neglected in this regard (Dunlop & Watson, 2022; Milotic, 2022; Poulin, 2021). Parasite conservation is important because parasites play important roles in ecosystems (i.e., they have a huge role in community structuring and even in pollutant sequestration) and they face a double threat (anthropogenic changes and possible co‐extinction with their hosts) that makes them highly susceptible to extinction (Carlson et al., 2017; Dobson et al., 2008; Dougherty et al., 2016; Kwak et al., 2020; Milotic, 2022).

A parasite may be associated with different hosts at different life stages (Benesh et al., 2021; Blasco‐Costa & Poulin, 2017; Haverkost et al., 2010), and thus it might be challenging to determine which host or life stage is the major factor constraining their geographical distribution. For species with both parasitic and free‐living life stages, the presence of the definitive host might be the determinant of the species' distribution (Auld & Tinsley, 2015; Haverkost et al., 2010; Terui et al., 2017; Waltari & Perkins, 2010). Parasites often have very low dispersal abilities (Poulin, 2007) and they may disperse via the help from the definitive host (Ellis et al., 2015; Terui et al., 2017; Waltari & Perkins, 2010). Furthermore, host switch (Ellis et al., 2015; Galbreath et al., 2020) and the different phenology of the parasite when associated with different hosts (e.g., Chiu et al., 2016; Waltari & Perkins, 2010) may also change predictions of their distribution patterns.

Among parasites with complex life cycles, horsehair worms (phylum Nematomorpha) present unique characteristics: they are one of the few animal phyla (but see Giribet & Edgecombe, 2020) where all the described species are parasites (Bolek et al., 2015; Schmidt‐Rhaesa, 2012). Additionally, they switch between parasitic and free‐living lifestyles at different life stages and the same species can utilize different hosts. Furthermore, they usually have at least one intermediate host and a final host during the parasitic stages (Bolek et al., 2015; Poinar, 2008; Schmidt‐Rhaesa, 2012).

In this study, we evaluated the use of SDMs for estimating the niche similarity between a parasite and its hosts. We chose to focus on the most common horsehair worm in Taiwan, *Chordodes formosanus* (Chiu, 2017), because it is currently known from several definitive host species (mostly from the Taiwanese giant mantis *Titanodula formosana*: Chiu et al., 2011, 2015, 2016, 2017) and it can be sampled both directly from the hosts and during the free living stage (Chiu, 2017; M. De Vivo, pers. obs.). We predicted that, based on the current ecological knowledge on the species (Chiu, 2017), the giant Taiwanese mantis should be the main host and therefore it should show the highest level of niche overlap with the parasite when compared to other hosts (Waltari & Perkins, 2010). All the modeling procedures were done by making SDMs using Maxent (Phillips et al., 2006) implemented in ENMeval 2.0 (Kass et al., 2021). This software can be used with presence‐only data and a background dataset, and it performs well compared with other SDM algorithms (Phillips et al., 2017). We also evaluated the possible range of each species by making binary maps to uncover possible areas where the species have not been reported yet.

## 2 MATERIALS AND METHODS

### 2.1 Model species

We made our models using a parasite—the gordiid *C. formosanus* Chiu et al. (2011)—and three different definitive hosts (three mantises, order Mantodea): the Taiwanese giant mantis *T. formosana* (Giglio‐Tos, 1912, previously known as *Hierodula formosana*; Vermeersch, 2020), the Indochina mantis *Hierodula patellifera* Serville, 1839 and the Japanese boxer mantis *Acromantis japonica* Westwood, 1889.

We assumed that the Taiwanese giant mantis was the main host of *C. formosanus* in Taiwan, given that the emergence peak of the horsehair worm in Taiwan matches with the period in which this mantis species reaches the adult stage, but it does not match with the period in which the two other mantis hosts reach adulthood (Chiu, 2017; Chiu et al., 2016, 2017). Additionally, due to abnormalities in the cuticle and size of *C. formosanus* individuals that develop inside the Japanese boxer mantis and katydids, Chiu et al. (2017) concluded that those animals should not be regarded as a main host of the hairworm species. Nevertheless, we included the former host in our dataset to note its distribution overlap with *C. formosanus* and see if there was a difference from the one calculated by comparing the worm's distribution with the distributions of the two giant mantises (subfamily Hierodulinae).

Although *C. formosanus* needs freshwater bodies for breeding and for finding an intermediate host (Chiu et al., 2011), we believe that mantises are good proxies for the presence of the species. Specifically, the larvae of horsehair worms can survive up to 2 weeks before encysting inside the intermediate host, and the larva itself has poor dispersing ability (Chiu, 2017; Chiu et al., 2016). The intermediate host emerges from the water as an adult in around 1 month (Chiu et al., 2016; but see Doherty & Poulin, 2022 for possible phenological alterations). Furthermore, the free‐living adult worms can live approximately 2 months in the wild (Chiu et al., 2016). Because they can spend 2 weeks as encysted larva, 1 or 2 months inside the intermediate host, and 2 months at most as an adult in the water, we argue that the majority of their lifespan (which lasts around a year; Chiu, 2017) is associated with their final host. As a result, the final host, the mantis, can be a good proxy for the worm's existence. We acknowledge that rivers play an important role in the reproduction and larval development of the hairworms. Nevertheless, we suspect that rivers may not play an important role in the dispersal of hairworms in Taiwan because both the larvae and free‐living adults exhibit low dispersal ability by themselves (Chiu, 2017; Chiu et al., 2016). Furthermore, the orology of the island impacts the river network in a way that makes it challenging for nonflying species associated with freshwater to disperse (Shih et al., 2006).

We did not include the reported host katydids in our study, given the scarce number of observations of the involved taxa in Taiwan. Therefore, we cannot validate if and how their distributions overlap with the hairworm. However, it is assumed that their influence on *C. formosanus*'s distribution should be less impactful than the mantises, given the following considerations: they have been reported as hosts very few times; the helminths show abnormalities when they grow inside them; the emergence peak of adult horsehair worms does not match the emergence of the adult katydids (Chiu et al., 2017).

### 2.2 Presence points

Presence data in Taiwan for the parasite and hosts were downloaded from “Research Grade” iNaturalist observations (which are defined as observations in which at least two‐thirds of the users who tried to identify the animal agreed on the species ID) on May 25, 2022.

In the case of *C. formosanus* and the two giant mantises, additional points were taken from local sightings, news, literature, and personal collection data. Horsehair worms are notoriously difficult to sample (Schmidt‐Rhaesa, 2012), but it is possible to observe some of them inside their hosts when they are developed and ready to emerge for reproduction (Bolek et al., 2015). *C. formosanus* can be seen at the abdomen tip of its hosts when it is ready to emerge (Chiu, 2017), and it is therefore possible to sample the hosts to collect the worm. Moreover, our personal collection data also include worms sampled as free‐living in the adult stage. Additionally, some of the *C. formosanus* points from iNaturalist were taken even if they were not “Research Grade”; although species identification in Nematomorpha can be extremely difficult from pictures alone (Bolek et al., 2015; Schmidt‐Rhaesa, 2012), we considered an observation to be *C. formosanus* if one of the three considered hosts were nearby in the pictures or if the worm clearly resembled a typical *Chordodes* specimen (i.e., presence of the leopard pattern as described by Schmidt‐Rhaesa, 2012), given that no other species of the same genus is known from Taiwan (Chiu, 2017).

In the case of the Japanese boxer mantis, given the uncertainty about the taxonomy of *Acromantis* in Taiwan (Chih‐Ting Hsu, pers. comm.), we decided to download all the available observations of said genus from the island and consider them as reports of a single species.

To avoid overfitting the data with multiple presence points in the same grids and to remove potential issues caused by sampling bias (Kramer‐Schadt et al., 2013), we thinned the data points using the R package “spThin”’ (Aiello‐Lammens et al., 2015), with each point set at least 10 km apart, one maximum possible output file and 100 repetitions to get an optimal number of presence points (Aiello‐Lammens et al., 2015). Some presence points for the two giant mantises were removed from the dataset due to the lack of data and environmental variables. In the end, 18 presence points were retrieved for *C*. *formosanus*, 78 for the Taiwanese giant mantis, 100 for the Indochina mantis, and 32 for the Japanese boxer mantis.

### 2.3 Environmental variables

We downloaded the 19 bioclimatic layers, which were adapted from the originals hosted on the website WorldClim2 (Fick & Hijmans, 2017), from Lin (2020), which is the Dryad archive of the data used for Chang et al. (2021). More precisely, these data were obtained from the original study by cropping the original layers to fit them to the Taiwan area. All the variables are at 30 s (~1 km^2^) resolution. We chose nonhighly correlated variables for each species. The decision was based on calculating a variance inflation factor maximum of 5 using vifstep() implemented in the “usdm” R package (Naimi et al., 2014), after getting the values of each variable from each presence point with the extract() function of the “raster” package (Hijmans, 2021). The chosen variables for each species are available in Supplementary Material 1 in Appendi S1.

### 2.4 Species distribution models

All the models were run using Maxent (Phillips et al., 2006) implemented in ENMeval 2.0 (Kass et al., 2021). Maxent applies maximum entropy distribution (defined as “closest to geographically uniform” or “most spread out, or closest to uniform”) to find the estimated distribution or habitat suitability of a species, given a set of variables (categorical and/or continuous) and presence points, together with background points that are considered an attempt to represent the environments in the study region (Phillips et al., 2006, 2017). The models for the hosts were run with 2500 background points randomly selected from each environmental predictor variables' stack, “linear, linear + quadratic, linear + product, linear + quadratic + product, linear + quadratic + hinge and hinge” (L, LQ, LP, LQP, LQH, H) as possible class features, nine (from 1 to 5, with a 0.5 interval) regularization multipliers, and four block partitions based on the spatial partitioning technique described in Radosavljevic and Anderson (2014) for spatial validation.

In the case of the parasite, given the low number of presence points available, we decided to run the models with an “*n* − 1 jackknife” partition method instead of block ones (as shown by Muscarella et al., 2014 for small datasets), using a bigger range of regularization multipliers (from 0.25 to 5, with an interval of 0.25 each) and added further class features, also evaluating “quadratic + product, quadratic + hinge, quadratic + threshold, linear + quadratic + product + threshold and linear + quadratic + hinge + product + threshold” and thus reaching a total of 11 class features (L, LQ, LP, QP, LQP, LQH, QH, H, QT, LQPT, LQHPT). Also, given that the majority of the points for *C. formosanus* were in the northern area of the island, we made a raster to account for the bias and then sampled the background points from it following indications from a GitHub discussion made by the developers of ENMEval (https://github.com/jamiemkass/ENMeval/issues/26; Supplementary Material 2 in Appendix S1).

### 2.5 Model evaluation and selection

There are several statistics that can be used to evaluate the goodness of a model. However, some of them might be inefficient in some cases, that is, measures based on thresholding might be based on a wrongly assumed population density or some others might need absence data that are not always available (Leroy et al., 2018; Merow et al., 2013). MaxEnt uses the area under the receiver–operator curve (AUC) as the default parameter for model evaluation (Phillips et al., 2006). AUC measurement is threshold independent and thus can be useful in case a threshold is not set, although there are some caveats to this. For example, it can penalize predictions beyond presence locations, which may cause issues with under‐sampled species (Merow et al., 2013).

Previously, the most frequently used statistics for evaluating SDMs made by the ENMeval package was the lowest corrected Akaike information criterion score (AIC_c_), which is a measure of both goodness‐of‐fit and complexity of the models computed by the package itself (Muscarella et al., 2014). However, this metric does not evaluate models from their validation or testing. Therefore, it hinders the possibility of true model evaluation (Kass et al., 2021). Given these considerations, we preferred to evaluate the models using the 10% omission rate (“or.10p.avg”) as the primary criterion and we chose models with values ≤0.1. Then, among these we selected the model with the highest average validation AUC (“auc.val.avg”, formerly known as AUCtest; Kass et al., 2021), which is based on “predicted values for the test localities (i.e. localities withheld during model training), averaged over k iterations” (Muscarella et al., 2014).

### 2.6 Binary maps

To get an insight into the predicted distribution of the species, we produced binary maps. The maps were thresholded according to the 10th percentile training presence (P10), using a custom script (Morrow, 2019; Supplementary Material 2 in Appendix S1). We decided to use P10 because it tends to work well with species with a low number of presence points and, although it tends to be more restrictive than other thresholds, it can also be useful for predicting potential areas in which the species may be present (Pearson et al., 2007; Radosavljevic & Anderson, 2014).

### 2.7 Niche overlap

We calculated the overlaps for the selected models using five metrics: Schoener's *D* (Schoener, 1968); the *I* statistic (Warren et al., 2008); relative rank (RR; Warren & Seifert, 2011); Pearson correlation coefficient (*r*; Warren et al., 2011); and the rank correlation coefficient rho, also known as Spearman rank correlation (Spearman, 1904).

Conventionally, Schoener's *D* and *I* are the most commonly used indices for evaluating niche overlap (Bates et al., 2020; Filz & Schmitt, 2015; Fourcade et al., 2022; Maher et al., 2010; Waltari & Perkins, 2010). Said statistics are computed by standardizing suitability to reach a sum of 1 over the measured geographic space. Conversely, RR checks the probability that two habitat patches have the same suitability from different models, the Pearson correlation coefficient evaluates linear correlations among variables (Warren, 2018; Warren et al., 2011), and rho assesses possible monotonic relationships (Spearman, 1904). In general, rho is a reliable metric for evaluating a species' response to the environment, while *D* and *I* are usually used to evaluate similarities in habitat suitability between results given a set of conditions (Warren, 2018; Warren et al., 2021). *D* and *I* do not consider if there is correlation among different models, but only if there is similarity between them, while rho does the opposite (Warren, 2018). *D* and *I* check if two or more species will interact in the geographic space, while rho emphasizes differences in the response to the predictor variables (Warren, 2018). RR can be regarded as similar to *D* and *I*, but it actually considers the quality differences in suitability estimates and it does not standardize said suitabilities for reaching 1 (Warren et al., 2011). The metrics were calculated using the Perl script ENMTools 1.4.4 (Warren et al., 2010) and its complementary R package (Warren et al., 2021).

## 3 RESULTS

### 3.1 Species distribution models and binary maps

The models selected according to the parameters discussed in the previous sections all had average validation AUC values >0.7 (ranging from 0.7155 to 0.759,), which indicates that the models had both better accuracy than random ones and were able to distinguish presences from absences (Table 1, Supplementary Material 3 in Appendix S1). Furthermore, the average 10% omission rate tended to be equal to or less than 0.1 for the mantises, although it was slightly higher for *C. formosanus* (Table 1, Supplementary Material 3 in Appendix S1), probably because of the relatively low number of presence points available.

**TABLE 1:** Data for selected SDMs, rounded up to the third decimal number.

Species	Tune arguments	AUC_val_	Or10_avg_
*Chordodes formosanus*	Rm 2.25 LQPT	0.759	0.111
Japanese boxing mantis	Rm 1.5 LQH	0.735	0.031
Taiwanese giant mantis	Rm 3.5 H	0.75	0.051
Indochina mantis	Rm 4.5 H	0.717	0.1

*Note*: Other metrics for the models are available in Supplementary Material 2 in Appendix S1. The models' raster files are available in Supplementary Material 3 in Appendix S1. AUCval, average validation AUC; H, hinge features; L, linear features; Or10avg, 10% training omission rates; P, product features; Q, quadratic features; Rm, regularization multiplier; T, threshold features.

**FIGURE 1:**
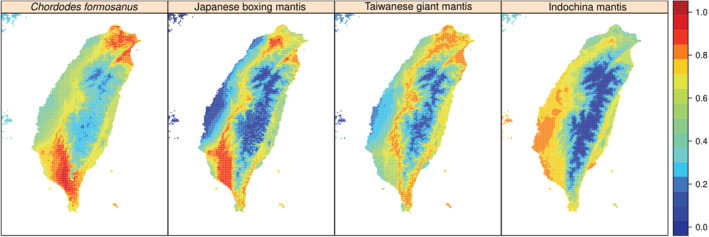
Selected suitability models. Highest values indicate better predicted suitability.

**FIGURE 2:**
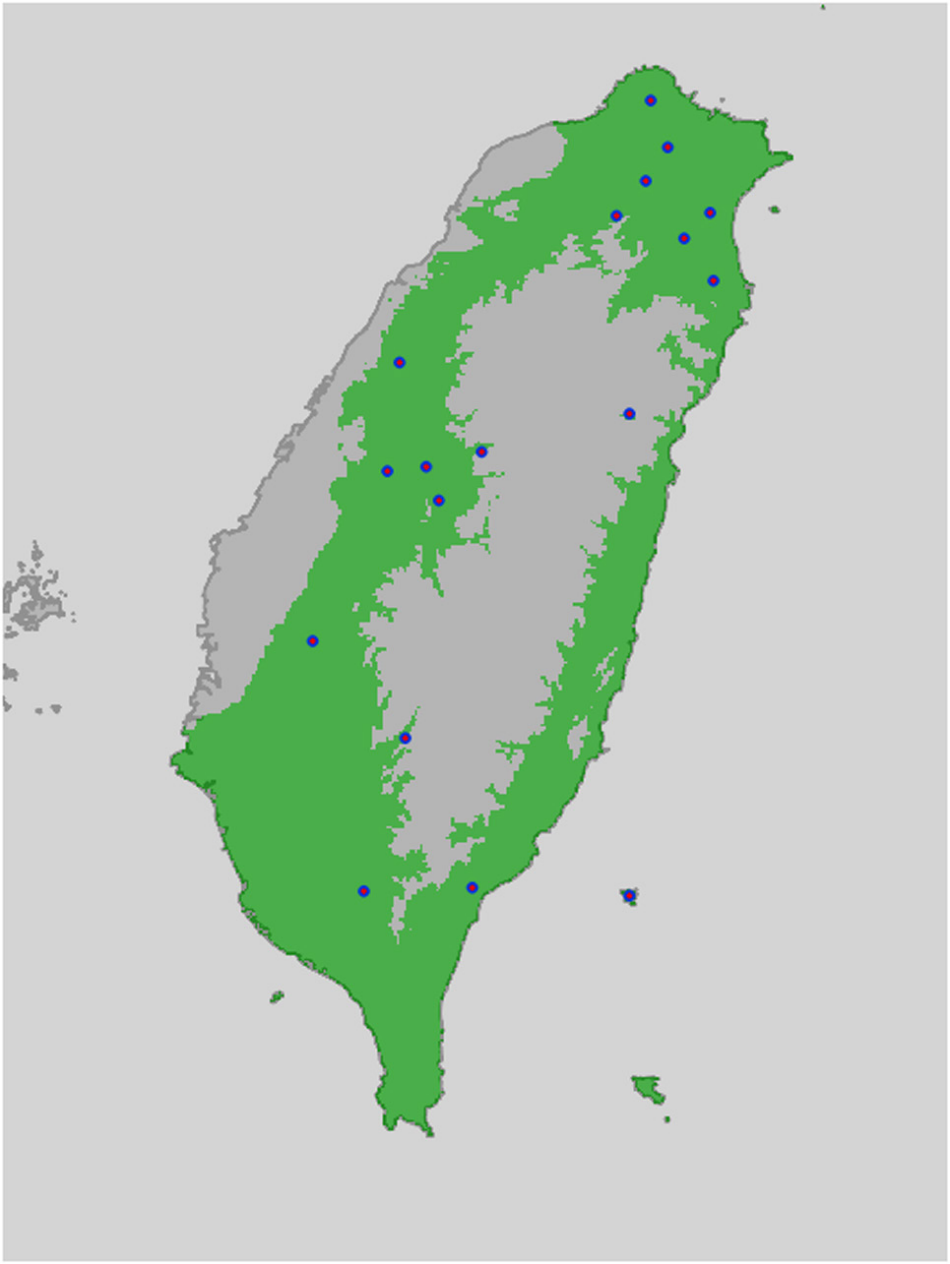
Binary range map for *Chordodes formosanus*, with the presence points used for modeling. The green area represents where the animal should be present.

According to the selected models, all the evaluated species tend to live in lowland areas (Figure 1), which is consistent with the literature about them. Binary range maps also show suitability outside of Taiwan for the Indochina mantis, which agrees with the fact this species is common in various areas of East and South‐East Asia (Supplementary Material 2 in Appendix S1). Several areas in which *C. formosanus* is not reported are regarded as suitable by the binary map (Figure 2).

### 3.2 Niche overlap

According to Schoener's *D* and *I* statistics, *C. formosanus* and the Taiwanese giant mantis's habitat suitability models were the most similar. According to RR, *r* and rho, the Japanese boxer mantis' distribution was the most similar to that of the parasite (Table 2; Supplementary Material 4 in Appendix S1).

**TABLE 2:** Used metrics and highest models' overlap, rounded up to the third decimal number.

Metric	Highest overlap	Value
Schoener's *D* (*D*)	*C. formosanus*‐Taiwanese giant mantis	0.903
*I* statistics (*I*)	*C. formosanus*‐Taiwanese giant mantis	0.993
Relative Rank (RR)	*C. formosanus*‐Japanese boxing mantis	0.86
Pearson correlation coefficient (*r*)	*C. formosanus*‐Japanese boxing mantis	0.891
Rank correlation coefficient (rho)	*C. formosanus*‐Japanese boxing mantis	0.899

*Note*: The other comparisons are available in Supplementary Material 4 in Appendix S1.

Abbreviation: *C. formosanus*, *Chordodes formosanus*.

## 4 DISCUSSION

### 4.1 Species distribution models and binary maps

Both the models (Figure 1) and the binary maps (Figure 2; Supplementary Material 2 in Appendix S1) highlight how the evaluated species tend to avoid the central high elevation mountain range in Taiwan, which is consistent with our current understanding of their ecology (Chiu, 2017). Interestingly, the binary maps produced by thresholding the models with the 10th percentile training presence highlights suitable areas for *C. formosanus* in which the species has not been reported yet (Figure 2).

**FIGURE 3:**
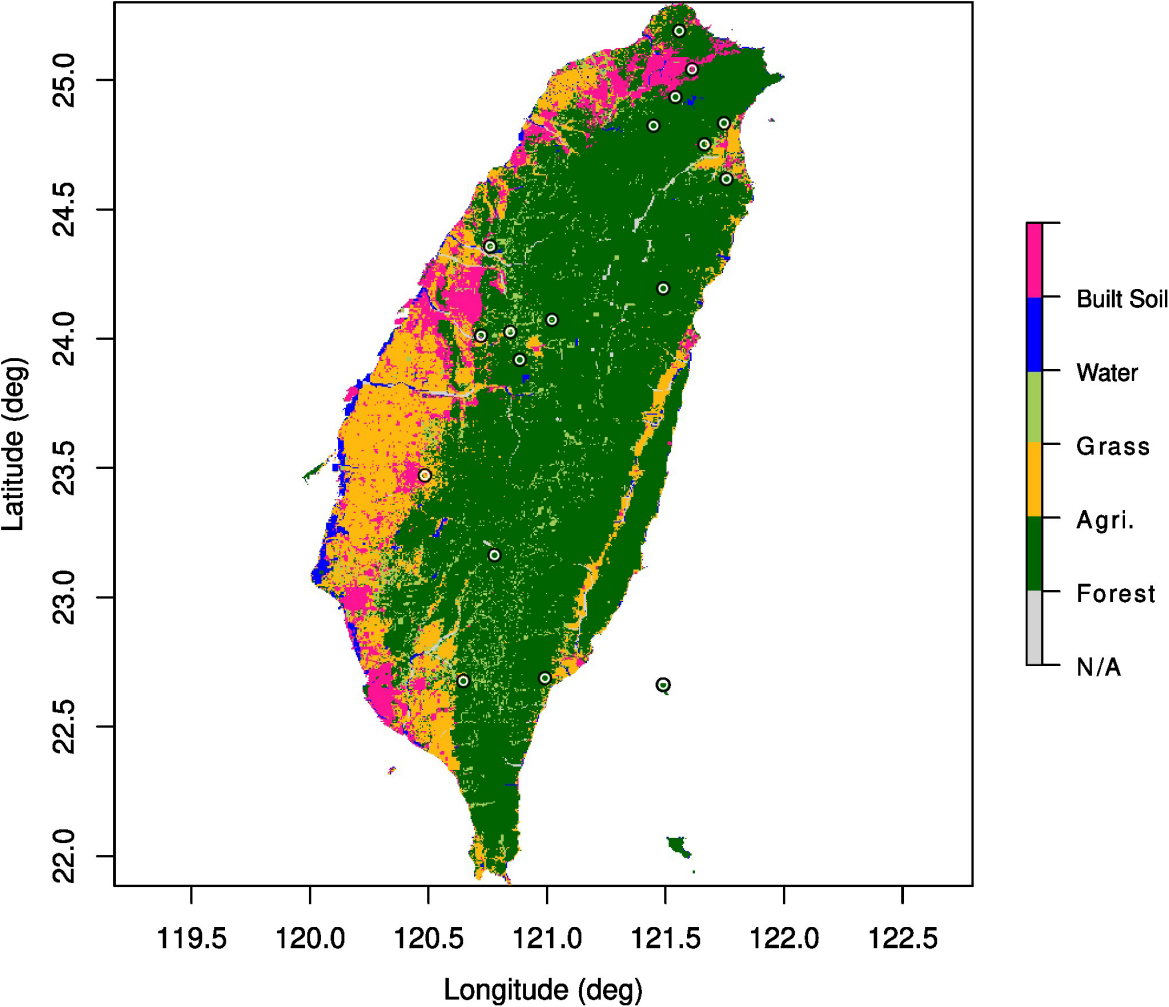
Land use types in Taiwan in 2015, plotted with the presence points used for modeling *Chordodes formosanus*. Data and scripts from Chen et al. (2019).

The conservation status of *C. formosanus*, like the one of other parasites in several animal clades or gordiids (Poinar, 2008; Schmidt‐Rhaesa, 2012), is not known (only two parasitic metazoans are listed in the IUCN Red List and around 460 macroparasite species have their risk of extinction assessed, while at least 3%–5% of the helminths parasitizing vertebrates are regarded at risk: Carlson et al., 2017, 2020; Dobson et al., 2008; Kwak et al., 2020), even though the species appears to be abundant in northern Taiwan (Chiu, 2017). However, it is known that hairworms can go through extirpation due to human intervention (Sato et al., 2014) and that they may be sensitive to several human‐related changes, such as clear‐cut logging (Sato et al., 2014), stream remediation (Chiu et al., 2016) and chemical pollution (Achiorno et al., 2018; Poinar, 2008). Taiwan has experienced a population growth (from 2.5 million inhabitants in the early 20th century to 23 millions in 2015) that has heavily influenced its land use, due to the need for agricultural and urbanized areas. Although forest areas started to recover after the 1980s, a subsequent reduction of inland waters also occurred (Chen et al., 2019). Given the sensibility of hairworms to human intervention, it is possible that populations were extirpated in heavily exploited areas (e.g., farmed areas) and that might explain the lack of reports. In particular, during the years of 1949–1956, there was an increase of agricultural land in the central and eastern areas of Taiwan. During the years of 1994–2015, the cultivated area decreased, except for the border region between central and southern area (Chen et al., 2019); those heavily agricultural areas are the ones where the reports of *C. formosanus* are lower or lacking (Figure 3). However, given the difficulties in sampling hairworms and the lack of standardized protocols for collecting them (Bolek et al., 2015; Schmidt‐Rhaesa, 2012), coupled with the fact that the human population of Taiwan is mostly concentrated in the northern area (Chen et al., 2019), it is possible that there might be a huge sampling effort bias. Bias in geographic sampling effort also happened in other parasitic taxa (Pappalardo et al., 2020) where people simply did not collect or report the species in some areas. Our model provides a projection for where future sampling efforts should be done, as shown in a study on ticks (Glass et al., 2021).

### 4.2 Niche overlap

Three of the metrics compiled by ENMTools found that the Japanese boxer mantis had the highest overlap with *C. formosanus*, while the other metrics found more similarity between *C. formosanus* and the giant Taiwanese mantis (Table 2; Supplementary Material 4 in Appedix S1). The difference among the results may be due to the different ways the metrics were calculated (see the Section 2).

Nevertheless, there is a high degree of overlap among hosts and the parasite (Supplementary Material 4 in Appedix S1), which may explain the latter's distribution (Waltari & Perkins, 2010). All four evaluated species are usually found at low elevation areas, especially in forest habitats (Chiu, 2017; Chiu et al., 2016). Therefore, it makes sense that the degree of overlap is high, although *C. formosanus* occupies freshwater bodies as an adult, like other species of the same family (Chordodidae) during their free‐living stage (Schmidt‐Rhaesa, 2012). Furthermore, *D* and *I* evaluate the interaction in the geographic space (Warren et al., 2021) and theoretically the difference between the two indices should be minimal (Warren, 2018; Warren et al., 2008). Additionally, *D* and *I* indices have often been used in empirical studies for showing overlaps between hosts and parasites (Filz & Schmitt, 2015; Maher et al., 2010; Waltari & Perkins, 2010) and for comparing distributions between different species (e.g., Fourcade et al., 2022). Following these metrics, we should say that the Taiwanese giant mantis generally interacts more with *C. formosanus* than the other hosts, which is consistent with the literature (Chiu, 2017; Chiu et al., 2011, 2016, 2017) and that implies this mantis is the main host, given the fact that a higher level of overlap should represent a higher level of dependency on that host (Waltari & Perkins, 2010).

It is surprising how much overlap there is between the boxing mantis and the worm, given that it contradicts Chiu et al. (2017), in which the authors argued that this mantis species may be an accidental host for *C. formosanus* due to the few reports and the fact that the peak in the number of adult worms does not occur at the same time as the peak of the emergence of said mantis. These results may suggest that the Japanese boxing mantis is more important as a host than previously thought. However, it must be noted that, in horsehair worms, host size influences the adult length (Ni et al., 2021); the latter is known to positively influence the fertility in these animals (Hanelt, 2009). *C. formosanus* individuals from Japanese boxing mantises tend to be smaller in size than that of those from Taiwanese giant mantises (Chiu et al., 2017; M. De Vivo, pers. obs.). Therefore, future studies addressing the differences in fecundity among *C. formosanus* from different hosts are required to understand whether the high niche overlap between hosts and parasite is caused by habitat similarity or by equal fitness exhibited by worms from different mantis species.

The degree of overlap among the Indochina mantis and all the other species tends to be lower compared to the ones among all the other evaluated taxa (Supplementary Material 4 in Appendix S1), probably because it is present in a wider range of environments and geographic areas compared to the other considered taxa. For example, it tends to be more common in urban environments compared to the other two considered mantises (Battiston et al., 2020; Chiu, 2017). However, this may change in Japan, in which the Indochina mantis is regarded by previous studies as the main host for *C. formosanus*, given that all the reported specimens of this hairworm species in Japan were exclusively associated with this mantis species (Chiu et al., 2011). Moreover, the Taiwanese giant mantis is not present in the Japanese islands (Vermeersch, 2020), and the Japanese boxing mantis seems to be absent in some areas and is not currently reported as a host in the country. Our hypothesis can be tested by making a separated SDM for *C. formosanus* and the Indochina mantis from Japanese records while excluding the presence data from Taiwan and then evaluating niche similarity. There are currently only a few presence points (around a total of 8, 3 of which are in a 10‐km area) for *C. formosanus* from Japan (Chiu et al., 2011), and this number might be insufficient for building a reliable SDM (van Proosdij et al., 2016). A comparative study in the future when more occurrence data become available will undoubtedly help us understand how exactly different factors affect niche overlap and the impacts on the predicted distribution of the parasites.

### 4.3 Ecological and modeling implications

Parasites are a very important part of their ecosystems, but they are often neglected in conservation plans (Carlson et al., 2020; Gómez & Nichols, 2013). Their complex life cycles or multiple hosts can pose serious issues for sampling (Gómez & Nichols, 2013). Therefore, prioritizing host sampling to find the parasites can be helpful. In other systems, the prey–predator interaction can be one of the main factors shaping animal distribution and diversity (Chang et al., 2021; Gusmão et al., 2020; Preuss & Padial, 2021). However, as mentioned before, the distribution of parasites can be fully dependent on the dispersal ability of the hosts due to the parasite's poor dispersal ability (Terui et al., 2017; Waltari & Perkins, 2010). It is still unclear how much the definitive hosts of *C. formosanus* influence its distribution, but the huge habitat similarity suggests that they tend to occupy the same geographical space. The geology of Taiwan may prevent gene flow among flightless animals (Shih et al., 2006). Therefore, flying hosts might serve as dispersal vectors for hairworms on the island. Previous field collections of mantises have been successful for also sampling *C. formosanus* in Taiwan (Chiu et al., 2011, 2015, 2017), indicating that the predicted geographical overlaps between the hosts and parasites have a biological significance.

Alternatively, intermediate hosts may aid the dispersal of *C. formosanus* in Taiwan. In the past, hairworm surveys were carried out mostly by sampling cysts inside intermediate hosts or “dead end” ones (i.e., Chiu et al., 2016; Harkins et al., 2016). The range of intermediate hosts for most parasites is unknown and some parasite species may even have multiple intermediate hosts during their development (Benesh et al., 2021; Blasco‐Costa & Poulin, 2017). The knowledge on the intermediate host range of *C. formosanus* is still incomplete (Chiu, 2017), like that of other hairworms (Hanelt & Janovy Jr., 2003). Some hairworm species exhibit low specificity for intermediate hosts (Schmidt‐Rhaesa, 2012), but the larvae do not infect all of them with the same intensity (Doherty et al., 2019; Hanelt & Janovy Jr., 2003). Additionally, the known range of *C. formosanus* includes the caddisfly *Chimarra formosana*, stoneflies in the genus *Kamimuria* and various Chironomidae midges. There seems to be a preference for species from the dipteran family (Chiu, 2017; Chiu et al., 2016), but there is currently no distributional data for these intermediate host species in Taiwan. The dispersal abilities of midges, stoneflies, and caddisflies can be very clade‐specific (Arce et al., 2021; Ferrington, 2008) and making the inference about their dispersal and distribution can be extremely difficult. Several questions will be challenging but necessary for future studies using SDM to answer:

1. how to incorporate the impacts of different hosts when studying parasite SDMs, and
2. how to make biologically informed hypotheses regarding the geographic distribution of the parasites and their overlap with their host at different life stages.

We lack knowledge on several aspects of hairworm biology and ecology due to a lack of natural history studies. In general, horsehair worms are overlooked because they do not infect humans or animals related to anthropic activities (i.e., pets and livestock; Bolek et al., 2015; Poinar, 2008; Schmidt‐Rhaesa, 2012) and cases of pseudo parasitism on mammals are very rare (Schmidt‐Rhaesa, 2012). However, gordiids are known to indirectly influence the overall food network by manipulating their hosts (i.e., crickets and katydids) to jump into freshwater, making the latter easy prey for predators like salmons and trouts. This leads to less predation by fish on benthic invertebrates, which in turn changes the nutrients' ratio in the freshwater due to an increased nutrient uptake by said benthic organisms (Sato et al., 2008, 2011, 2012). Furthermore, hairworms' biodiversity is severely understudied and some areas in the world have more described taxa than areas where the known diversity should be higher (Poinar, 2008). As a result, we may never find undescribed taxa because they go extinct, like what has happened in other clades (Tedesco et al., 2014). Therefore, understanding the most important factors that determine the distribution of hairworms and modeling the species' potential distributions is fundamental for understanding their ecology and possible extirpation events in the future.

## 5 CONCLUSIONS

Previous studies on host–parasite analyses focused on using D and I as indices to evaluate their niche and geographical overlaps (Filz & Schmitt, 2015; Maher et al., 2010; Waltari & Perkins, 2010). These metrics only evaluate similarity between probabilities of distribution between two raster files while disregarding possible different responses to the same environment (Warren, 2018). However, parasites may have different niche dimensions when associated with different hosts (Waltari & Perkins, 2010). Future studies testing the response of different host and parasite species to different environmental factors and if other metrics can better explain their distribution patterns are needed. Moreover, understanding the host range of parasites can be useful for sampling design. For example, in the only other SDM project related to hairworms to our knowledge, Harkins et al. (2016) were able to sample horsehair worms by modeling the distribution of freshwater snails (which are “dead‐end hosts” for Gordiida) and sampled several hairworm species in the predicted areas. Our results and the previous study (Harkins et al., 2016) showed the potential of SDM for designing sampling for parasite conservation studies.

## AUTHOR CONTRIBUTIONS

**Mattia De Vivo:** Conceptualization (equal); formal analysis (lead); writing – original draft (lead); writing – review and editing (equal). **Jen‐Pan Huang:** Conceptualization (equal); supervision (lead); writing – review and editing (equal).

## Supporting information


**Appendix S1**.

## Data Availability

The cropped bioclim layers used in this study are available from Lin (2020). The processed presence points, the scripts and the results of the analyses are available in the Supplementary Materials from Zenodo (https://zenodo.org/record/6899278, https://doi.org/10.5281/zenodo.6899278).
